# Genome Sequence of a Novel Reassortant and Very Virulent Strain of Infectious Bursal Disease Virus

**DOI:** 10.1128/genomeA.00730-17

**Published:** 2017-08-24

**Authors:** Hyun-Jeong Lee, Il Jang, Sun-Hwa Shin, Hee-Soo Lee, Kang-Seuk Choi

**Affiliations:** Avian Disease Research Division, Animal and Plant Quarantine Agency, Gimcheon, Republic of Korea

## Abstract

Here, we report the complete coding genome sequence of a novel reassortant and very virulent infectious bursal disease virus (IBDV), designated JBN2011. Characterization of the JBN2011 genome suggests that it is a rare recombinant virus having a very virulent IBDV segment A and a Bursine-2-like attenuated IBDV segment B.

## GENOME ANNOUNCEMENT

Infectious bursal disease virus (IBDV) is an important viral pathogen of poultry. Two serotypes of IBDV are recognized, serotype 1 viruses, which are pathogenic for chickens, and serotype 2 viruses, which are not. Serotype 1 viruses are further classified into the following four pathotypes: attenuated (at), classical virulent (cv), antigenic variant (av), and very virulent (vv) (1). IBDV is a birnavirus with a bisegmented RNA genome (segments A and B).

In 2011, in South Korea, the egg inoculation method was used to isolate an IBDV from pathognomonic bursas of Fabricius (BFs) from a dead 4-week-old broiler chicken suspected of having infectious bursal disease (2). The virus caused a mortality rate of 80% in 3-week-old pathogen-free chickens, indicating vvIBDV. Here, the complete coding genome sequence of the virus was obtained using direct sequencing of both directions and overlapping PCR. Editing and alignment of multiple sequences were carried out using CLC Genomics Workbench version 6.7.2 (CLC bio, Aarhus, Denmark). Phylogenetic analysis was performed using MEGA version 7.0 (3).

Segment A has a coding region (3,085 nucleotides) containing two partially overlapping open reading frames that encode VP5 (159 amino acids [aa]) and a precursor polyprotein (pp) (1,012 aa). During virus replication, the PP is cleaved to yield VP2, VP4, and VP3 by autocatalysis. The VP5 protein contains four extra N-terminal aa residues (MLSL), as found in vvIBDV and cvIBDV (4). VP2 contains several aa residues (222A, 242I, 253Q, 256I, 279 D, 284A, 294I, and 299S), which are unique among vvIBDV strains (5, 6). Phylogenetic analysis based on the nucleotide sequences of VP2 showed that it clustered with vvIBDV strains. These findings indicate that JBN2011 harbors segment A derived from field vvIBDV.

Segment B encoding VP1 (881 aa) harbored aa substitutions at positions I61V, D146E, N147G, E242D, A287T, M390L, D393E, P562S, P687S, and R695K, which are typical of non-vvIBDVs (7–10). In particular, the aa sequence of the B-marker region (11) of VP1 was identical to that of Bursine-2 (atIBDV vaccine strain) and Edgar (cell culture-adapted cvIBDV). Other vaccine strains, such as Winterfield 2512, Lukert, D78, and 228E, differ from JBN2011 with respect to the aa at position 121; they also show lower nucleotide identity (99.2 to 97.0%) with JBN2011 than with Bursine-2 and Edgar (99.8%). This is supported by results of phylogenetic analysis of VP1 sequences; JBN2011 was most closely related to Bursine-2-like atIBDVs. Altogether, these findings indicate that JBN2011 harbors segment B derived from atIBDV vaccine strains, such as Bursine-2, but still maintain a vv phenotype. The results also highlight the potential risk of using live IBDV vaccines in regions of endemicity.

### Accession number(s).

The sequence of JBN2011 has been deposited in GenBank under accession numbers MF188862 (segment A) and MF188863 (segment B).
